# Genomic Selection in Aquaculture: Application, Limitations and Opportunities With Special Reference to Marine Shrimp and Pearl Oysters

**DOI:** 10.3389/fgene.2018.00693

**Published:** 2019-01-23

**Authors:** Kyall R. Zenger, Mehar S. Khatkar, David B. Jones, Nima Khalilisamani, Dean R. Jerry, Herman W. Raadsma

**Affiliations:** ^1^Centre for Sustainable Tropical Fisheries and Aquaculture, College of Science and Engineering, James Cook University, Townsville, QLD, Australia; ^2^ARC Research Hub for Advanced Prawn Breeding, James Cook University, Townsville, QLD, Australia; ^3^Sydney School of Veterinary Science, Faculty of Science, The University of Sydney, Camden, NSW, Australia; ^4^Tropical Futures Institute, James Cook University Singapore, Singapore, Singapore

**Keywords:** aquaculture, animal breeding, genomic selection, genetic improvement, oyster and shrimp

## Abstract

Within aquaculture industries, selection based on genomic information (genomic selection) has the profound potential to change genetic improvement programs and production systems. Genomic selection exploits the use of realized genomic relationships among individuals and information from genome-wide markers in close linkage disequilibrium with genes of biological and economic importance. We discuss the technical advances, practical requirements, and commercial applications that have made genomic selection feasible in a range of aquaculture industries, with a particular focus on molluscs (pearl oysters, *Pinctada maxima*) and marine shrimp (*Litopenaeus vannamei* and *Penaeus monodon*). The use of low-cost genome sequencing has enabled cost-effective genotyping on a large scale and is of particular value for species without a reference genome or access to commercial genotyping arrays. We highlight the pitfalls and offer the solutions to the genotyping by sequencing approach and the building of appropriate genetic resources to undertake genomic selection from first-hand experience. We describe the potential to capture large-scale commercial phenotypes based on image analysis and artificial intelligence through machine learning, as inputs for calculation of genomic breeding values. The application of genomic selection over traditional aquatic breeding programs offers significant advantages through being able to accurately predict complex polygenic traits including disease resistance; increasing rates of genetic gain; minimizing inbreeding; and negating potential limiting effects of genotype by environment interactions. Further practical advantages of genomic selection through the use of large-scale communal mating and rearing systems are highlighted, as well as presenting rate-limiting steps that impact on attaining maximum benefits from adopting genomic selection. Genomic selection is now at the tipping point where commercial applications can be readily adopted and offer significant short- and long-term solutions to sustainable and profitable aquaculture industries.

## Introduction

Animal improvement programs are based on using phenotypic information of individuals in conjunction with knowledge on genetic relationships and quantitative genetic principles. Breeders have enhanced production traits of farmed species by selecting superior individuals as parents for succeeding generations. Aquaculture is the farming of aquatic organisms and is the fastest growing animal protein production sector globally, supplying approximately 50% of seafood in 2015 (Fishery Statistics, FAO, 2017). However, despite the rapid growth in aquaculture production, only ~10% is currently based on genetically improved animals (Gjedrem et al., 2012). This low percentage of improved animals under culture is due to several factors, including the size and maturity of industries, the inability to domesticate or control reproduction in many species, the large number of species farmed, difficulties in retaining pedigree throughout the entire production process, inability to collect large phenotypic data sets, and a general lack of informative genetic parameter information for traits (Jerry et al., 2001; Gjedrem et al., 2012).

Within aquaculture breeding programs, the initial focus has been on growth, which is a moderately heritable trait and relatively easy to select. Here the industry achieved rates of genetic progress per generation 4–5 times greater than that realized in livestock (Gjerde and Korsvoll, 1999). For aquaculture species that have been improved, such as Atlantic salmon (*Salmo salar*), Nile tilapia (*Oreochromis niloticus*), and the Pacific white shrimp (*Litopenaeus vannamei*), selection for growth has dramatically increased efficiencies helping establish these species as global commodities. While growth is one of the important determinants of aquaculture productivity, other traits such as disease resistance, feed conversion efficiency, environmental tolerance, and carcass or product quality are also significant. These later traits are typically harder to select for as they are difficult-to-measure, can often only be measured late in life, involve destructive sampling, or have low heritability (e.g., Yáñez et al., 2015). Additionally, the estimation of breeding values (EBVs) for breeding candidates themselves often cannot be estimated for these traits based on individual phenotypes, but rather they are estimated based on phenotypic records and EBV of their siblings (termed sib selection). Sib selection therefore only utilizes the between-family genetic variance within a population and ignores half of the available genetic variance (i.e., ignores the within-family genetic variance; see Hill, 2013). Reduced phenotypic variance and increased generation intervals to measure close relatives for lifelong performance traits invariably result in sub-optimal genetic gain.

The development of species-specific breeding objectives is based on economic goals of the industry and a comprehensive understanding of the genetic architecture of the traits. Within silver lipped pearl oyster *Pinctada maxima* breeding programs, host oyster shell size, donor oyster pearl quality traits (i.e., size, weight, color, luster, and complexion), and disease resistance have all been identified as important phenotypes for selection based on their economic value and genetic basis (Kuchel et al., 2011; Jerry et al., 2012; Jones et al., 2014a,b). However, due to difficulties in obtaining accurate on-farm animal performance and relationship data, selective breeding progress of these traits has been somewhat limited (e.g., Jones et al., 2017a). For marine shrimp (i.e., white-legged shrimp *L. vannamei* and black tiger shrimp *Penaeus monodon*), phenotypes such as animal morphology (i.e., size, weight, color, and carapace dimensions), fecundity, disease resistance, and/or environmental robustness have been identified by industry as important traits to ensure ongoing commercial productivity (e.g., Cock et al., 2009; Castillo-Juárez et al., 2015). Here the challenge is again obtaining robust on-farm animal performance and relatedness data, which then can be used to calculate accurate environment-specific breeding values.

### General Principles of Genomic Selection

With the realization that traditional breeding programs for traits other than growth are difficult to implement, alternative approaches to estimate the genetic merit of breeding individuals of these traits have been explored. In particular, rapid developments in genomics and quantitative analytical methods have resulted in breeders incorporating genetic marker technology in the form of marker-assisted selection (MAS) to aid animal selection. Although this technique can be useful for some traits where quantitative trait loci (QTL) of large effect have been identified, application of MAS to improve complex traits controlled by many genes of smaller effect is limited. Genetic improvement in these traits can only be achieved through more advanced genomic-based methods, where it is now possible to accurately predict genome-wide molecular breeding values for improved animal selection (Eggen, 2012). This approach, termed genomic selection, first proposed by Meuwissen et al. (2001), has gained significant application within the animal and plant breeding communities. In this approach, decisions on selecting breeding candidates are derived from genomic breeding values predicted from genome-wide loci (VanRaden, 2008).

Genomic selection is based on the theory that with sufficient high numbers of loci across the genome, most quantitative trait loci will be in strong linkage disequilibrium with at least one marker (Meuwissen et al., 2001). As such, genomic selection simultaneously estimates the combined genetic effects of all relevant QTL and provides accurate predictions of genetic merit for a trait. Furthermore, genome-wide markers are directly used to compute the genomic relationship matrix (GRM), which can then be used to compute genomic estimated breeding values (GEBVs) using genomic best linear unbiased prediction methods (i.e., GBLUP, VanRaden, 2008; Legarra et al., 2009; Aguilar et al., 2011; Goddard et al., 2011). GRM, even based on a smaller subset of markers, can provide an accurate estimate of the proportion of the genome shared by related individuals and hence provides higher accuracy of estimation of breeding values as compared to estimates based on pedigree information alone (Habier et al., 2007; Forni et al., 2011; Vallejo et al., 2017). However, GBLUP accuracy is also reliant on other parameters including range of genome linkage disequilibrium (LD), training population size (Daetwyler et al., 2008; Andonov et al., 2017), relationship between training and validation data sets (Meuwissen et al., 2001), heritability of the trait (Daetwyler et al., 2008), and genetic architecture of the trait, including the size of allele substitution effects at QTL (Meuwissen et al., 2001; Goddard, 2009).

Following on from GBLUP, a single-step GBLUP method (ssGBLUP) was developed (Legarra et al., 2009; Aguilar et al., 2010) to utilize all available information. This method simultaneously uses all phenotypic, genotypic, and pedigree information, including trait information on non-genotyped individuals, thus increasing genomic prediction accuracy (e.g., Aguilar et al., 2011). Both the GBLUP and ssGBLUP methods generally assume that all SNPs (and genomic regions) have the same weight and variance. However, this is not entirely accurate, and alternate genomic evaluation methods such as Bayesian methods (i.e., BayesA, BayesB and BayesC; Meuwissen et al., 2001; Habier et al., 2011; Gianola, 2013), weighted GBLUP and ssGBLUP (WGBLUP and WssGBLUP, respectively; Snelling et al., 2011; Tiezzi and Maltecca, 2015), and trait-specific marker-derived relationship matrix (TABLUP; Zhang et al., 2010) have been developed to take *a priori* information such as the presence of major genes or QTL that affect the trait of interest into account. These methods can set alternate weights to SNPs that are in high LD with a causal mutation or associated with QTL with a relatively large effect, which improves accuracy of predicting GEBV.

**Figure 1:**
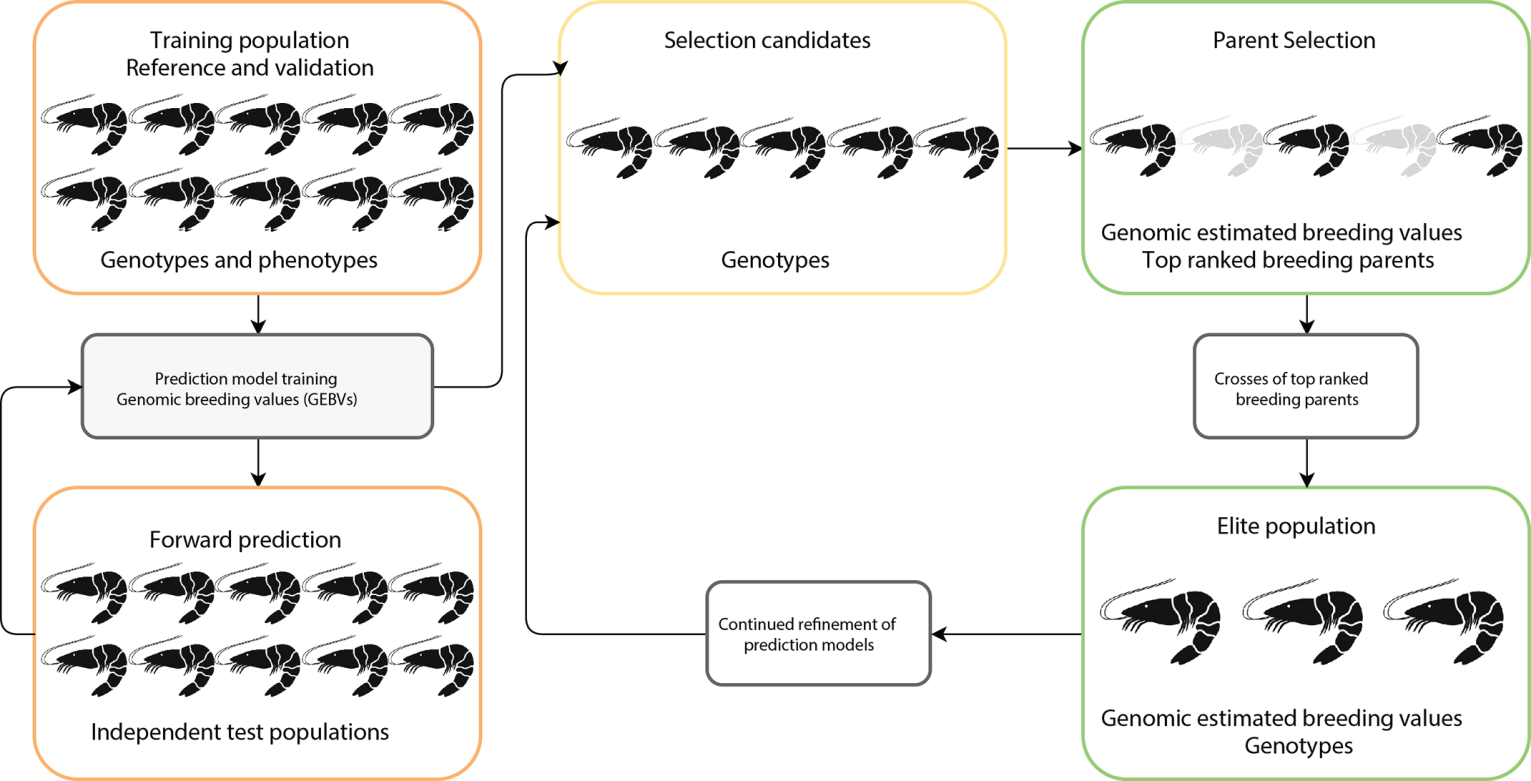
Schematic representation of genomic selection approach in aquaculture. Implementation consists of optimizing prediction equations in a reference population (i.e., farm breeding population), with large numbers of individuals, which have genotype and phenotype information. The prediction equations are then validated on independent test animals (i.e., related generations to reference population). Once the prediction equations are fully optimized, the prediction method is applied to selection candidates to select superior replacement broodstock.

The basic principles for implementing genomic selection (see Figure 1) involve a training population in which animals are phenotyped and genotyped, and SNP effects estimated using a range of statistical models (Taylor, 2014). This typically involves splitting the training population into reference and validation data sets to test performance of the statistical models (Eggen, 2012). Following this, the predictive SNP effects are then independently validated in a related test population (independent of the training population) and checked for accuracy of prediction against highly reliable estimated breeding values (EBV). Once the prediction equation has been fully developed, the appropriate genomic selection method is applied to a group of new selection candidates with GEBV data generated for each animal and the best animal selected for breeding. The prediction equation for the GEBV is under constant refinement as breeding programs progress.

### Applications of Genomic Selection to Aquaculture

The application of genomic selection can significantly improve the genetic response of breeding programs in aquaculture. Initial theoretical investigations of genomic selection in aquatic species suggest that a higher accuracy of selection and subsequently higher rates of genetic gain (up to 10% for body weight) can be achieved compared to traditional selection (Campos-Montes et al., 2013; Ødegård et al., 2014b; Castillo-Juárez et al., 2015). Additional improvements in the rate of genetic gain can also be made by reducing generation interval by selecting candidates early in life based on their genomic breeding value (Campos-Montes et al., 2013; Castillo-Juárez et al., 2015). Furthermore, genomic selection can reduce rates of inbreeding by up to 81% when compared with traditional selection programs (Vandeputte and Haffray, 2014). This is particularly important for aquaculture species where fecundity can be extremely high, potentially limiting on-farm effective population size (Ne), leading to loss of diversity and/or inbreeding (Dupont-Nivet et al., 2006; Holtsmark et al., 2008). Within aquaculture, genomic selection has the profound potential to change the breeding structure from single-line mating systems to multi-family breeding programs in communal rearing environments, decreasing infrastructure requirements and potentially optimizing genetic diversity and gain (Fernández et al., 2014). Further efficiency of breeding programs can be captured through structured management of genotype by environment interactions and incorporating dominance and epistatic effects (Dupont-Nivet et al., 2008; Nilsson et al., 2016). Finally, genomic selection can help track signatures of artificial selection and genetic diversity in the process of domestication of farmed species (López et al., 2014; Yáñez et al., 2015).

The success of the practical implementation of genomic selection in aquaculture is through well-designed breeding programs negating genotype by environment interactions (Ibarra and Famula, 2008; Rauw and Gomez-Raya, 2015) and common environmental and inbreeding effects (Bentsen and Olesen, 2002; Havenstein et al., 2003; Hely et al., 2013). The foundation of such programs requires effective genomic resources, understanding trait genotype to phenotype relationships, accurate phenotypic recording systems at an industrial scale, and appropriate mating designs to optimize genetic gain (Gjedrem et al., 2012). Each of these aspects can have different challenges depending on the specific aquaculture species and production system. In this review, we provide an overview of the requirements and challenges of implementing genomic selection, with a particular emphasis on shrimp and pearl oyster industries.

## Pathway for Incorporation of Genomic Selection into Aquaculture Breeding Programs

### Building Genomic Resources

Single-nucleotide polymorphisms (SNPs) have become the marker of choice in genetics research due to their high abundance, co-dominant inheritance, relative ease of high-throughput discovery, and low cost of genotyping per locus. The use of microarray technology has been a feasible choice for large-scale SNP genotyping across terrestrial and crop production industries (Fan et al., 2010; Rasheed et al., 2017). As a result, there has been increasing interest from researchers and industry in the development of medium-density (1,000–10,000 s) to high-density (>100,000 s) solid-state SNP genotyping arrays for aquaculture species. Although the value of SNP genotyping arrays has been widely recognized for genetic map development, association studies, genomic selection predictions, and population genetic studies (Abdelrahman et al., 2017; Guppy et al., 2018), there are only a handful of aquaculture species that have off-the-shelf commercially available arrays listed (e.g., catfish, oyster, salmon, and trout – Affymetrix Axiom, Santa Clara, CA, USA; shrimp – Illumina Infinium, San Diego, CA, USA), or previously custom built SNP genotyping arrays developed (Table 1). The lack of commercially available genotyping SNP arrays for the majority of aquaculture species adds additional cost to genetic investigations, as these resources need to be first developed and tested. Furthermore, comprehensive genome sequence assembly projects required for large-scale SNP identification, and array probe design is limited among many aquaculture species (Guppy et al., 2018).

**Table 1:** Development of medium- to high-density SNP microarrays used in aquaculture species.

Species	Number of array SNPs	SNPs utilized	Platform technology	References
*Atlantic salmon*	286,021	135,682	Affymetrix Axiom	Houston et al. (2014)
	200,000	159,509	Affymetrix Axiom	Yáñez et al. (2016)
	55,000	47,070	Affymetrix Axiom	Bangera et al. (2018)
	5,919	5,918	Illumina Infinium	Lien et al. (2011)
*Catfish*	250,113	200,860	Affymetrix Axiom	Liu et al. (2014)
	693,567	535,618	Affymetrix Axiom	Zeng et al. (2017)
*Coho salmon*	220,001	189,501	Affymetrix Axiom	Martine et al. (2018)
*Common carp*	250,000	185,150	Affymetrix Axiom	Xu et al. (2014)
*European oyster*	14,950	11,151	Affymetrix Axiom	Gutierrez et al. (2017)
*Giant tiger shrimp*	6,000	4,237	Illumina Infinium	Baranski et al. (2014)
*Nile tilapia*	58,466	40,190	Affymetrix Axiom	Joshi et al. (2018)
*Pacific oyster*	1,536	1,172	Illumina GoldenGate	Hedgecock et al. (2015)
	190,420	133,984	Affymetrix Axiom	Qi et al. (2017)
	40,625	27,697	Affymetrix Axiom	Gutierrez et al. (2017)
*Pacific-white shrimp*	8,967	6,941	Illumina Infinium	Jones et al. (2017b)
*Rainbow trout*	57,501	49,468	Affymetrix Axiom	Palti et al. (2015)
*Silver-lipped pearl oyster*	2,782	1,343	Illumina Infinium	Jones et al. (2013)

Owing to the recent advances in next-generation sequencing (NGS), high-throughput genotyping-by-sequencing (GBS) technologies, which detect and genotype SNPs through whole or reduced genome sequencing simultaneously, have significantly reduced both the cost of developing and genotyping SNPs for non-model species (e.g., Elshire et al., 2011; Andrews et al., 2016). As such, GBS is rapidly becoming one of the SNP genotyping methods of choice for aquaculture species (see Robledo et al., 2017), either by directly providing genotype data or by discovering markers for the design of solid-state SNP arrays. However, compared to SNP array-based genotyping platforms, GBS requires significantly more quality control (QC) measures to ensure robust genotype data. This is primarily a result of the molecular technique itself, which can introduce spurious and missing data without rigorous QC and data filtering methods. Erroneous GBS data could then result in incorrect biological conclusions, such as unreliable inferences about individual/population diversity and relatedness (Gorjanc et al., 2015; Andrews et al., 2016). Genotyping errors in GBS data are often derived from multiple factors, including sequencing base-call errors, sequence alignment/clustering errors, and null alleles from low-coverage sequencing or mutations in the restriction enzyme binding site in library preparations (e.g., Nielsen et al., 2012; Bryc et al., 2013; Davey et al., 2013; Ilut et al., 2014; Sims et al., 2014; Mastretta-Yanes et al., 2015; Verdu et al., 2016). Aquaculture species can be particularly vulnerable to these GBS errors as many species genomes are highly polymorphic and repetitive, which can inflate erroneous genotype data based on the aforementioned factors. This is a problem particularly observed for crustaceans and oysters (e.g., Lal et al., 2016; Abdelrahman et al., 2017; Yuan et al., 2017).

There are currently a number of methods and software available for detecting aberrant GBS data and for improving the reliability and accuracy of genetic calculations used in genomic studies using GBS. Methods for the detection and exclusion of aberrant SNP data are based on the elimination of GBS loci that contain too many SNPs, deviate from Hardy-Weinberg or Mendelian inheritance, have too high or too low read coverage for specific allele/genotype combinations, or are revealed by software capable of detecting existence of paralogous loci (Catchen et al., 2013; Gayral et al., 2013; Bianco et al., 2014; Eaton, 2014; Lexer et al., 2014; Torkamaneh et al., 2017). Apart from ensuring the highest quality sequence reads (i.e., QScore ≥ 30), a commonly used method to discard possible sequencing errors in GBS data sets consists in the elimination of very low minor allele frequency SNPs (i.e., 1/50 allele ratio), particularly when not observed among multiple individuals or populations (Roesti et al., 2012; Lal et al., 2016). Overall, the QC and filtering methods used should be carefully chosen based on the error rate of the sequencing method, the read depth obtained, the assembly and SNP calling method used, and the repeat complexity and polymorphism level of the genome studied (Schatz et al., 2010; Sims et al., 2014). Currently, the impact of cumulative GBS errors on genomic selection accuracy is not fully understood.

The outcome of robust GBS data filtering is ultimately the reduction of downstream data analysis errors, but the approach also comes at a cost of reducing the overall number of available SNPs and/or individuals (when achieving higher sequence depth), or increasing costs to achieve a required threshold (Kim et al., 2016). To address this issue, recent methods have been developed to accurately impute low confidence or missing SNPs among individuals based on comprehensive GBS metric data (e.g., Money et al., 2015; Chan et al., 2016; Brouard et al., 2017). Furthermore, this approach can also be incorporated into downstream genomic analyses to improve the reliability and accuracy of computational outcomes. For example, improvements in the calculation accuracy of unbiased estimates of relatedness (i.e., GRMs) for genomic selection applications were obtained when accounting for sequence read depth of genotype calls within different mathematical models (Dodds et al., 2015; Cericola et al., 2018). The improvements are gained through correcting for individual/genotype sequence depth and standardizing SNP density and quality. A similar approach has also been applied in a novel genetic linkage mapping method, whereby improved confidence in placement of loci was obtained by incorporating GBS read depth information, improved filtering capability, and using a statistical approach to model and correct errors (Bilton et al., 2018). The ability to maximize GBS data and generate accurate computational outcomes is especially important for aquaculture species that often have genetic resource limitations. Based on the continued interest in this area, it is highly likely that approaches to fully utilize GBS data (particularly in aquaculture species) will continue to be developed, or at least until high-density solid-state SNP genotyping arrays become more accessible and cost-effective.

### Optimizing Use of Genomic Resources

**Figure 2:**
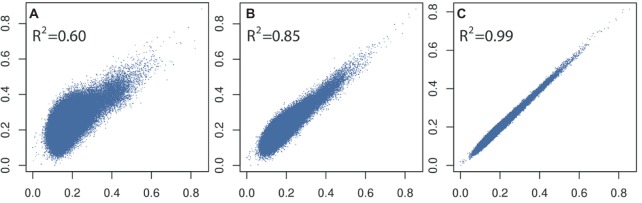
Comparison and correlations of SNP-based kinship estimates (rG) **(A)** 96 versus 7,500 SNPs, **(B)** 384 versus 7,500 SNPs, and **(C)** 3,000 versus 7,500 SNPs calculated on 1,000 *L. vannamei* samples.

In general, a large training population is required for accurate genomic prediction, particularly for traits with low heritability (Goddard et al., 2011). However, most commercial breeding stocks in aquaculture have small effective population size meaning a moderate size training population would be sufficient. Apart from optimizing the number of training or selection candidates for routine genotyping (i.e., based on genomic selection modeling and farm breeding scheme, e.g., Sonesson and Meuwissen, 2009), reducing the cost or number of genome-wide markers is a viable solution. Our own data using shrimp and pearl oysters (Jones et al., 2017a; Khatkar et al., 2017) show that derivation of accurate genomic relationships can be achieved with relatively low-density SNP panels (~3,000 SNPs; Figure 2; Zenger et al., 2017), compared to those derived from medium- to high-density SNP panels (e.g., ~50,000+ SNPs; see also Ødegård et al., 2014a). This result has also been observed in Pacific-white shrimp (3.2 k SNPs; Wang et al., 2017) and Atlantic salmon (5 k SNPs; Tsai et al., 2015) investigations. However, such accuracies deteriorate rapidly if very low-density SNP panels are used (<1,000 SNPs). In addition, a larger panel of SNPs is required for accurately estimating lower degree of relationships present in an outbred population.

Another method to reduce the cost of genotyping is through imputation of genotypes, where most of the animals can be genotyped with a low-cost, low-density SNP panel. The genotypes of these animals can be imputed up to high density by using information on a smaller number of reference individuals (typically broodstock) genotyped with a larger high-density SNP panel that also captures the same SNP as represented on smaller arrays. Such imputed *in silico* genotypes can then be used for genomic selection and other genomic analyses. Such strategies have been shown to improve the accuracy of genomic selection in livestock (Khatkar et al., 2012) and aquaculture species (Tsai et al., 2017). However, the accuracy of imputed genotypes is critical for efficient use of these low-density panels. The accuracy of imputation is affected by a number of factors including proportion of markers to be imputed, relatedness between reference and imputed test individuals (Hickey et al., 2012; Khatkar et al., 2012), correct ordering of markers on the genome map, local pattern of linkage disequilibrium and minor allelic frequency (e.g., Hickey et al., 2012; Badke et al., 2013), and number of individuals represented in the reference set (Druet et al., 2010).

The required number of individuals in the reference panel and number of markers in the low-density panel depends on the effective population size of the breeding stock and relationship between reference and test populations. A small effective population size, as present in many aquaculture stocks, will require smaller number of animals in the reference panel and can be imputed with high accuracy with smaller number of SNPs in the low-density panel. Moreover, if all the contributing broodstocks are genotyped with the high-density panel, the accuracy of imputation in the progeny, genotyped with even smaller SNP panel, could be quite high using a pedigree-based imputation approach (Hickey et al., 2012).

There are limited number of studies into imputation accuracy and its application to aquaculture breeding programs. Kijas et al. (2017) reported high accuracy (0.89–0.97) of imputing genotypes from a low-density panel (0.5–5 K) to a high-density panel (78 K) using a multi-generation reference population in farmed Tasmanian Atlantic salmon. Similarly, Tsai et al. (2017) showed that an accuracy of imputation of 0.90 could be achieved using very low-density panels in farmed Atlantic salmon using two-generation pedigree data sets. However, accurate imputation requires knowledge about the precise location of SNPs across the genome. For most aquaculture species, genetic linkage maps and/or genome assemblies are in the early stages of development (Abdelrahman et al., 2017).

### Linking Genotype to Phenotype – Genes of Large Effect

Industrial-scale selective breeding programs in aquaculture commenced in the 1960s with the development of Atlantic salmon breeding programs in Norway. Today, selective breeding programs have been implemented in over 60 species of fish and shellfish (Gjedrem and Baranski, 2009; Abdelrahman et al., 2017; Houston, 2017). Following traditional phenotypic or genetic selection (i.e., EBVs), the integration of genetic markers into MAS breeding programs was expected to increase the genetic response observed by increasing the efficiency and accuracy of selection (Laghari et al., 2014; Yue, 2014; Abdelrahman et al., 2017). However, for a MAS breeding program, utility is limited to detect genes of large effect with closely linked loci. These gene effects are typically detected through QTL linkage mapping and association studies.

QTL detection is the first step toward identifying causative genes and polymorphisms that contribute directly to the variation observed within a trait. However, very few examples of causative genes have been identified. In practice, QTL mapping is a powerful approach, where large family groups are clearly defined even if marker density is low. In contrast, since association testing exploits linkage disequilibrium, it is more suitable for large outbred populations with small family sizes and requires high marker density (Witte, 2010). The development of high-density SNP genotyping arrays in aquaculture has led to an increased uptake of genome-wide association studies (GWASs), including Atlantic salmon (Tsai et al., 2015), rainbow trout *Oncorhynchus mykiss* (Reis et al., 2018), catfish *Ictalurus punctatus* (Zhou et al., 2017), and common carp *Cyprinus carpio* (Zheng et al., 2016).

Increasing growth and body size has been a major goal of many aquaculture selective breeding programs due to its ease of measure and moderate-to-high heritability (Gjedrem, 2000; Gjedrem and Baranski, 2009). As a result, much research effort has been invested in QTL mapping, whereby QTL have been detected for these complex traits, for example, the rainbow trout (Wringe et al., 2010), Asian *Lates calcarifer*, European sea bass *Dicentrarchus labrax* (Massault et al., 2010; Wang et al., 2015), Atlantic salmon (Reid et al., 2004; Baranski et al., 2010; Gutierrez et al., 2012; Tsai et al., 2015), common carp (Laghari et al., 2015), scallop *Argopecten irradians* (Li et al., 2012), Pacific oyster *Crassostrea gigas* (Guo et al., 2012), pearl oyster *P. maxima* (Jones et al., 2014a,b), shrimp (Baranski et al., 2014; Abdelrahman et al., 2017), and many others. However, these QTL only explain a small proportion of the total additive genetic variation, and the validation of these QTL is limited, with only a few being confirmed in multiple independent families or populations.

Another high priority for aquaculture breeding programs is delaying time to sexual maturation in several species since it is energetically expensive and precocious maturation can impair growth and reduces meat quality (Thorpe, 1994; Küttner et al., 2011). As a result, QTL analysis for sexual maturity has been carried out in many species including the rainbow trout (Haidle et al., 2008; Easton et al., 2011), Atlantic salmon (Pedersen et al., 2013; Gutierrez et al., 2014a,b), and Atlantic char *Salvelinus alpinus* (Moghadam et al., 2007; Küttner et al., 2011). One major QTL for sexual maturation in the Atlantic salmon was found independently by two studies (Ayllon et al., 2015; Barson et al., 2015). Both studies report that these QTL explain from 33 to 39% of the phenotypic variation and are likely controlled by the gene VGLL3 (Ayllon et al., 2015; Barson et al., 2015). Finally, disease outbreaks are a major problem for the culture of aquatic species, and therefore, identifying disease resistant stock has been a major goal of breeding programs. Disease challenge testing is often utilized to measure the response to a disease within a species by recording survival of animals within known pedigrees. Such studies have reported moderate-to-high heritabilities indicating that there is high potential for improvement of disease resistance through breeding programs (reviewed in Jørgen et al., 2011). However, a recent review by Guppy et al. (2018) concluded that although QTL studies in *L. vannamei* and *P. monodon* provided a valuable insight into the architecture of disease survival and tolerance traits, they failed to provide the necessary information to apply findings through commercial MAS programs.

One successful MAS disease resistance case study has been reported on infectious pancreatic necrosis (IPN) resistance in Atlantic salmon, where major QTL explain more than 29% of the observed variance (Moen et al., 2009; Houston et al., 2010). The incorporation of these QTL within selective breeding programs resulted in a reduction in IPN from 47% in 2009 to 7% in 2010 within freshwater populations (Moen et al., 2009; Houston et al., 2012) and has now been demonstrated as a successful means of controlling the disease (Moen et al., 2015). A second case study is on the Lymphocytis disease in the Japanese flounder *Paralichthys olivaceus*, whereby one QTL, inherited in a dominant Mendelian fashion, accounted for over 50% of the total phenotypic variance associated with survival to the Lymphocytis disease (Fuji et al., 2006; Sakamoto et al., 2006; Ozaki et al., 2012). QTL mapping for disease resistance has also been conducted in the eastern oyster *Crassostrea virginica* for MSX and Dermo (Yu and Guo, 2006), the European flat oyster *Ostrea edulis* for Bonamiosis (Lallias et al., 2009), and the Atlantic salmon for salmonid alphavirus (Gonen et al., 2015), ISAv (Moen et al., 2007), and *Gyrodactylus salaris* parasitic disease (Gilbey et al., 2006; for review, see Yáñez et al., 2014).

Since the proliferation of QTL analysis in aquaculture species and even with documented success cases, MAS programs have only been successful for relatively simple traits where major QTL have been identified (Yue, 2014). Most traits of economic importance in aquaculture species are proving themselves to be polygenic and often have low-to-moderate heritabilities. As a result, these QTL studies searching for specific loci may be of limited commercial value in the era of genomic selection. Furthermore, the benefit of incorporating *a priori* weighted SNP effects of these QTL into genomic selection methods needs to be fully evaluated.

### From MAS to Genomic Selection

Currently, the reported implementation of genomic selection in commercial aquaculture is still in its early days and has been limited to a handful of high-value species (i.e., rainbow trout, Abdelrahman et al., 2017; Atlantic salmon, Bangera et al., 2018; and the Tasmanian Atlantic salmon strain, Verbyla et al., 2018). However, a number of examples of demonstrating accuracy of genomic prediction across a range of traits and other species in aquaculture species have been recently published, for example, sea lice resistance in Atlantic salmon (Tsai et al., 2016, 2017), bacterial cold-water disease resistance in rainbow trout (Vallejo et al., 2017, 2018), pasteurellosis resistance in gilthead sea bream *Sparus aurata* (Palaiokostas et al., 2016), shell size in Yesso scallops *Patinopecten yessoensis* (Dou et al., 2016), Greenshell mussel *Perna canaliculus* (Ashby et al., 2018), body weight and meat quality in large yellow croaker *Larimichthys crocea* (Dong et al., 2016) and channel catfish (Garcia et al., 2018), growth traits of Pacific white shrimp (Wang et al., 2017), resistance to viral nervous necrosis in European sea bass (Palaiokostas et al., 2018a), juvenile growth rate in common carp (Palaiokostas et al., 2018b), resistance against *Piscirickettsia salmonis* in a farmed Atlantic and coho salmon *Oncorhynchus kisutch* population (Barría et al., 2018), resistance against *P. salmonis* and infectious pancreatic necrosis virus in rainbow trout (Yoshida et al., 2018), growth traits in yellowtail kingfish *Seriola lalandi* (Nguyen et al., 2018), and resistance to amoebic gill disease in Atlantic salmon (Robledo et al., 2018). The accuracy of genomic prediction reported in the aforementioned studies varies from 0.16 to 0.83 (median = 0.60) for various disease survival traits and from 0.3 to 0.8 (median = 0.6) for the growth and body size-related traits. The accuracy of genomic predictions was higher (i.e., 155%, Barría et al., 2018; and 11%, Yoshida et al., 2018) than those based solely on pedigree information. However, most of these studies implemented cross validation with test data sets derived from randomly separating validation individuals from training populations and are thus near identical in distribution (i.e., mirror predictions). Experience from other species suggests that the accuracy represented by a mirror prediction is substantially higher than a forward prediction (i.e., candidates under selection; Moser et al., 2009). Nevertheless, the levels of accuracies observed in these initial investigations demonstrate potentially for applying genomic selection in breeding schemes in aquaculture species. Within our own current selective breeding research programs, genomic selection has been evaluated for direct integration into shrimp breeding programs for multiple production traits (e.g., size, disease resistance, color, and survival; Khatkar et al., 2017), as well as pearl oyster breeding programs for both host oyster and donor oyster traits (e.g., shell size and pearl quality traits; Jones et al., 2017a).

### Accurate and Low-Cost Phenotyping

Accurate phenotypes on commercially important traits are critical for any breeding program and particularly in the case of genomic selection. This becomes especially challenging in aquaculture, where large numbers of animals need to be recorded. Any error in the trait recording will reduce effective estimated heritability and hence realized genetic gain. High-throughput and precise phenotyping strategies are required to supply the large amount of trait data required for commercial scale genomic selection applications. Within this framework, the objective is to increase the accuracy, precision, and throughput of phenotypic assessment while reducing costs and minimizing labor in an intensive production system. Today, phenotyping is quickly emerging as the major operational bottleneck limiting the power and speed of commercial genomic selection programs (e.g., Cobb et al., 2013). This problem is compounded in aquaculture, where fecundity, progeny numbers from breeding pairs, and variable survival rates create circumstances, where individual phenotypes and traceability are nearly impossible to obtain without new methodologies. Furthermore, aquaculture does not have the benefit of standardized global phenotyping programs such as in livestock (e.g., dairy cattle). Designing effective on-farm phenotyping strategies requires integrated solutions incorporating biologists, computer scientists, statisticians, and engineers.

Over the last decade, mechanized automation, imaging, and software developments have paved the way for high-speed accurate data acquisition. Within these developments, digital imaging has emerged as a cornerstone to capturing high volumes of phenotypic information. Computer vision is an enabling technology and has already allowed many animal and plant production traits to be measured efficiently and accurately across different production industries, including aquaculture (i.e., McCarthy et al., 2010; Mathiassen et al., 2011; Cobb et al., 2013; Nasirahmadi et al., 2017; Saberioon et al., 2017). Aquaculture animal monitoring and evaluation with machine vision systems (MVSs) over the past several years have led to higher productivity and profitability through improved farm management practices and/or superior phenotype data collection used for selection strategies (see Mathiassen et al., 2011; Zion, 2012; Saberioon et al., 2017). MVS (2D or 3D imaging) in aquaculture has been extensively examined, and the accuracy between animal image analysis (i.e., fish, shrimp, oysters, and scallops) and phenotypic measurements (i.e., shape, size, volume, weight, color, and fillet quality) is very high (i.e., ≥ 0.95; see Odone et al., 2001; Harbitz, 2007; Pan et al., 2009; Zion, 2012; De Verdal et al., 2014; Hong et al., 2014; Zhang et al., 2014; Hao et al., 2015; Sture et al., 2016; Saberioon et al., 2017; Konovalov et al., 2018). When applying this technology to non-invasive automated on-farm flow-through MVS, fish length has accurately been estimated in the rainbow trout (Miranda and Romero, 2017), as with fish mass in Jade Perch (Viazzi et al., 2015) with low relative mean errors (5.2 and 6.0%, respectively). Furthermore, fish skin or fillet color and pearl quality traits (e.g., color, luster, and complexion), which are traditionally recorded as categorical traits, can now be recorded as highly reliable continuous quantitative traits based on ultraviolet-vis spectrophotometry measurements (e.g., Urban et al., 2013; Kustrin and Morton, 2015). The performance of MVS and traditional color measurements has been compared with Atlantic salmon, rainbow trout, and pearls with spectral patterns produced by MSV more representative and consistent of the real color (Yagiz et al., 2009; Mamangkey et al., 2010; Colihueque et al., 2011, 2017; Toyota and Nakauchi, 2013), which will ultimately improve genomic selection predictions.

Other emerging aquaculture phenotyping techniques are near infra-red (NIR) spectroscopy and hyperspectral imaging (HSI), which combines spectroscopy with imaging technology. These techniques are able to quantify and evaluate the chemical (e.g., fat, protein, and moisture) and physical (e.g., freshness, texture, and color) attributes of aquatic animals with relatively high accuracies of prediction (*r* > 0.8; see Liu et al., 2013; Saberioon et al., 2017). In HSI, the spectral reflectance of each pixel is acquired for a range of wavelengths including visual and NIR spectra. The resulting information is a set of pixel values at each wavelength in the form of an image, which can be correlated with traits with detailed spectral and spatial distribution information (ElMasry and Sun, 2010). All of these MVSs are able to extract and analyze quantitative information from digital images and have the ability to improve the accuracy of the phenotype by electronically analyzing the data at a pixel level across spectral regions not always visible to the human eye. More importantly, these technologies are advancing toward being fully non-invasive and automated, whereby animals need not be removed from their environment or sacrificed to obtain accurate phenotypic measurements. This not only improves farm productivity but also allows within-family selective breeding for traits that could only be recorded on siblings due to destructive sampling techniques.

MVS usually consists of two components, the image acquisition system hardware (i.e., UV-vis, NIR, and HIS) and data extraction system software. The latter typically incorporates computer-based processing and optimized statistical methods and algorithms specific for the trait of interest, which is often the limiting factor in applying MVS. The development of advanced image analysis software including artificial neural network (ANN) algorithms based on machine learning approaches has been an important step forward in the development of analysis systems for automated MVS phenotyping (e.g., Grys et al., 2017). Machine learning ANNs are devised of algorithms that broadly aim to mimic neural pathways in the human brain. By developing and selecting the correct architecture, ANNs can distinguish parameters of effect from inputs of noisy digital image data to outputs of the key signals of effect, to be used in regression, classification, clustering, reducing dimensionality, or detecting anomalies (Bishop, 2006; Schmidhuber, 2015). This is particularly important within industry settings, where MVS often needs to be implemented as a part of ongoing farm practices and is sometimes subject to non-uniformity in digital image recording.

**Figure 3:**
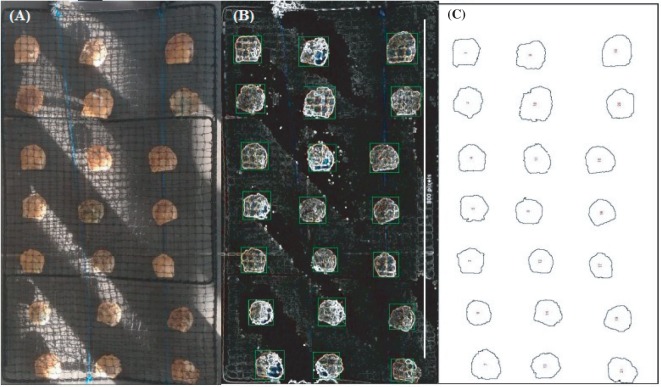
Different stages of predicting pearl oyster size through MVS and machine learning algorithm. Pearl oysters in net are placed on a table while being cleaned, and visual image is taken from above. **(A)** Oyster net image depicts one of the most challenging visual capture situations in pearl oyster commercial environment. **(B)** Oysters and net have low contrast from the background, and lighting is variable. **(C)** Sliding window CNNs correctly identified and measured oyster size with >96% accuracy. (Photo image by Preston Toole).

Within our own research programs (i.e., marine shrimp and pearl oyster), machine learning algorithms have allowed precise inexpensive phenotyping across diverse production traits. For example, body weight of black tiger shrimp *P. monodon* at harvest could be predicted from the high-throughput images with very high accuracy with a correlation coefficient between actual and predicted weight of 0.97 in the test animals using deep learning models. Similarly, MVSs have been used for pearl oyster growth data as well as pearl quality traits (e.g., color, size, luster, and completion). Although still in development, sliding window algorithms and convolutional neural network (CNN) with rule association-based clustering yielded high accuracy (exceeding 96%) in object character recognition for the pearl oysters in nets within the full spectrum of commercial situations (Figure 3; Zenger et al., 2017). By definition, CNN learning algorithms get more precise when presented with more data. This supervised learning approach has been undertaken with developing methodologies on how to automate the entry of commercial data into a NoSQL or graph-based database.

## Understanding Factors Affecting Utility of Genomic Selection

In aquaculture, genomic-based improvement programs can have a rapid impact on genetic improvement, particularly through the use of a structured nucleus-breeding scheme. As with traditional selective breeding programs, the potential of genomic selection will vary across different species depending on differences in life cycle, fecundity, effective population size, and breeding objectives. To our knowledge, there have been no empirical examples that compare commercial strategies to maximize rate of genetic gain using genomic information. Simulation studies provide a powerful alternative to experimental empirical estimates for parameters affecting responses in selective breeding programs in aquaculture, including inbreeding rates (Bentsen and Olesen, 2002; Dupont-Nivet et al., 2006; Sonesson et al., 2010, 2012; Skaarud et al., 2011, 2014), estimating the genetic gain (Sonesson and Meuwissen, 2009; Nirea et al., 2012b; Sonesson and Ødegård, 2016), and increasing the accuracy of selection (Nielsen et al., 2009; Vela-Avitúa et al., 2015). These general principles equally apply to selective breeding programs incorporating genomic selection. Consequently, we review the current state of knowledge in this area applied to aquaculture breeding programs. At present, 36 simulation studies based on traditional breeding strategies or genomic selection based have been conducted relevant to aquaculture of which most have predominantly focused on mating design, number and size of families, number of generations, genome size, marker density, and selection strategy. However, most of these studies were adopted through relatively simplistic approaches in breeding designs tackling few single parameters at a time and omitting complicated interactions between multiple parameters. Furthermore, parameters such as genotype by environment interactions, application of heterosis in multi-trait selection, realistic linkage disequilibrium, bio-economical modeling, and index selection have been largely ignored in simulation studies to date, yet warrant investigation in order to efficiently optimize potential breeding programs. An overview of different parameters and their influence on selective breeding programs including genomic selection are identified later.

### Mating Design

Choosing the right optimized mating strategy is one of the key components in any breeding design that could affect the rates of inbreeding and long-term genetic gain (Nirea et al., 2012a; Sonesson and Ødegård, 2016). Dupont-Nivet et al. (2006) compared single pair, nested design and factorial mating on genetic variability, inbreeding, and selection response. The results showed that a factorial mating strategy results in the lowest inbreeding and highest response to selection, while single pair mating was the worst option. Holtsmark et al. (2006) and Holtsmark et al. (2008) investigated different approaches through the combination of several sub-populations and random or structured diallel-cross mating in the base population while holding a fixed rate of inbreeding. The results showed that a diallel-cross mating within the base population followed by optimum contribution selection in subsequent generations can reduce the inbreeding to 0.5% per generation. Later, it was shown that application of “minimum co-ancestry and maximized ancestry co-variation mating” was even better in reducing inbreeding to roughly 0.004 per generation (Nirea et al., 2012a). However, variation in inbreeding coefficients and genetic gain across studies has been seen as a result of interactions between mating design (Nirea et al., 2012a) and effective population size (Dupont-Nivet et al., 2006).

### Number of Families

The number of families or breeders included within a breeding program can dramatically influence the trade-off between the rate of inbreeding or retention of genetic diversity and the selection intensity. Through the application of mass selection, Bentsen and Olesen (2002) highlighted that increasing the number of breeders from 4 to 100 in isolated lines can significantly reduce the rate of inbreeding across different heritabilities. It was also expected that the response to selection would increase at the same time, but significant improvements were not seen for the higher rates of heritability. It was further shown that increasing the number of families from 50 to 400 and applying the optimum contribution selection increased total genetic gain, while using 400 full-sib families kept the selection intensity at approximately 0.6 (Skaarud et al., 2014). Nonetheless, within genomic selection simulations, using less than 1,000 markers in combination with different family sizes (1, 10, and 100) did not result in meaningful reductions in the rate of inbreeding (Δ*F* = 0.019–0.011) (Sonesson et al., 2010). However, increasing the number of families from 100 to 1,000 in conjunction with 5,000 SNPs reduced inbreeding from 0.014 to 0.006, while it also substantially increased genetic gain (Δ*G* = 0.17–0.40) without any noticeable effect on the accuracy of selection (Sonesson and Ødegård, 2016). The large number of families resulted in higher genetic gains by increased selection intensities, whereas 5,000 SNPs allowed accurate prediction of breeding values. Overall, it seems that total number of 200 families in conjunction with optimum contribution selection could be a reasonable quantity in breeding designs (Skaarud et al., 2011, 2014).

### Size of Families

Most aquaculture species are highly fecund compared to livestock. The ability of aquaculture species to produce large numbers of progeny to satisfy commercial operations can result in reduced numbers of contributing families on farm, leading to reduced genetic progress and higher rates of inbreeding (Gjedrem and Baranski, 2009). Therefore, optimizing the contribution of families can significantly improve the efficiency of the breeding program. Sonesson (2005) applied optimum contribution selection with constraints on change in inbreeding and obtained 76–92% of genetic gains by selecting parents from only the top 100 genotyped selection candidates as compared to genotyping all individuals (1,000, 5,000, 10,000). Dupont-Nivet et al. (2006) could not obtain significant level of improvement of genetic gain by increasing family size from 20 to 200 progeny per family. Increasing the size of the families from 4,000 to 8,000 has also shown to be ineffective in capturing extra genetic gain (Nielsen et al., 2011; Sonesson and Ødegård, 2016). Skaarud et al. (2011) compared breeding designs with 8 to 200 progeny per family and showed genetic gain increased sharply by increasing family size to 50 with diminishing returns with further increases in family size, especially beyond 100. It appears that a family size of 50–100 progeny per family maybe optimal for minimizing inbreeding and maximizing genetic gain.

### Number of Generations

Utilizing simulation studies, the effect of altering the number of generations within aquaculture selective breeding programs has yet to be studied separately. However, it has been shown that both additive genetic variation and genetic gain could reduce over generations using selection based on pedigree information (Holtsmark et al., 2006, 2008). Although inclusion of genetic marker data improved the capture and retention of genetic gain over several generations through genomic selection simulations (Sonesson, 2007), it also increased inbreeding. This initial sub-optimal result was thought to be due to assumptions of low mating ratio (male/female: 1:2) and a relatively low number of QTL and markers. However, subsequent analysis using higher number of loci (1,000–5,000) in combination with greater population size (30–60) increased the accuracy of selection and genetic gain over five generations (Sonesson and Meuwissen, 2009). Beyond that, even using the combination of 10,000 loci and different QTL sizes (100, 400, and 2,000) did not really increase the genetic gain by great margins after 15 generations (Liu et al., 2015).

### Genome Marker Density and Total Number of Markers

Benefits of genomic selection using different SNP densities across different chromosomes have been evaluated over traditional means of estimating breeding values through the use of pedigree and phenotype information (Solberg et al., 2008; Sonesson and Meuwissen, 2009). The number of chromosomes used in different simulation studies ranged from 1 (Uleberg and Meuwissen, 2011) to 20 (Fernández et al., 2014; Meuwissen et al., 2014; Vela-Avitúa et al., 2015). In a major QTL simulation analysis, Sonesson (2007) showed that doubling the number of markers from two to four could increase the total genetic gain by approximately 2% in Generation 2 and 11% in Generation 3. Given the relative low allelic diversity of SNP markers, Solberg et al. (2008) concluded that almost three times as many SNP markers are needed compared to microsatellites to reach the same accuracy in genomic selection. Moreover, increasing the marker density eightfold (from 1N_e_/morgan to 8N_e_/morgan) can increase the accuracy of genomic selection from 0.69 to 0.86 (Solberg et al., 2008). They then concluded that using a family size of 100, at least 24,000 loci (100N_e_ for 30 morgan) are needed to obtain adequate selection accuracy. Lillehammer et al. (2013) later showed that even lower density of markers at 50–100 per Morgan can increase the accuracy of selection and genetic gain while reducing inbreeding, if the size of families is set to 200 across at least 100 families in the breeding program. They simply investigated the variation in marker density from 50 to 100 per Morgan, which resulted in increasing the accuracy of selection from 0.48 to 0.56 and raising the total genetic gain from 1.58 to 1.82, while at the same time, inbreeding was reduced from approximately 0.01 to 0.009.

#### Application of Stagewise and Index Selection

The application of multi-stage selection in traditional breeding programs was modeled in the simulation by Sonesson (2005). The study used the combination of walk-back and optimum contribution selection. This method resulted in a reduction in genotyping for parentage analysis while also maintaining genetic gains and avoiding an increase in inbreeding. This simulation was later expanded by Sonesson (2007) through using a lower number of markers and a simple breeding design. When applying these outcomes to genomic selection implementation on farm, the data suggest that total genotyping effort and hence cost can be greatly reduced by using a staged selection approach while capturing most if not all the benefits of genomic selection.

Index selection is relatively new in aquaculture simulation studies. When considering multi-trait selection, over- or under-weighted indices can result in sub-optimal profitability of the breeding program. Sánchez-Molano et al. (2016) investigated productivity and fitness traits based on different indexes (50/50, 75/25, and 100/0 index weights between the two objectives, respectively). The inbreeding coefficient in all scenarios increased over 20 generations using both pedigree and genomic information, but genomic selection was more efficient at reducing inbreeding and increasing genetic gain compared to pedigree-based selection. Most simulation studies to date have focused on simple trait analysis, while most commercial breeding applications are currently concentrating on multi-trait designs. Therefore, there is a need to expand genomic selection studies incorporating multi-trait breeding designs and capturing multi-trait genomic effects (i.e., pleiotropy) and genetic correlated effects at a genome level.

#### Exploiting Non-additive Genetic Variance

Most simulation studies have applied a simplistic additive model. The utilization of dominance and epistasis can potentially increase the power of genomic selection in cross-bred populations including family line crosses. Hence, both epistasis and dominance effects in addition to additive effects help to predict animal phenotypes more accurately. Besides, dominance and epistasis effects might also impact the additive genetic effect (Esfandyari et al., 2015). While Esfandyari et al. (2015) accounted for heterosis in cross-bred populations, the simulated genome size was quite small, which might have resulted in an overestimation of the accuracy of selection. Consequently, there is further requirement to expand simulation studies comparing efficiency of breeding design and incorporating non-additive components and possibility of developing lines with higher cross-bred performance.

#### Unequal Family Distribution and Maternal Effects

In traditional on-farm communal breeding designs, identification of families before selection is almost impossible, and using fixed family size is not realistic. For example, both shrimp and molluscs are highly prolific spawners with large variation between females and have unequal family sizes, and contribution is highly variable in commercial breeding programs (Khatkar et al., 2017). As such, unequal contribution, or over representation of a few highly fecund families, has the risk of increasing inbreeding, especially if the number of families is low. Therefore, accounting for unequal family contribution in simulation studies in order to avoid higher rates of inbreeding is warranted. Furthermore, in commercial breeding designs, some females produce most of the commercial progeny, and this puts an emphasis on investigating the importance and prediction of maternal effects through genomic selection, especially in aquatic systems where the number of breeders is low. No studies have considered these aspects in the application of genomic selection.

#### Use of Realistic Species-Specific Linkage Disequilibrium

Using genomic information, accuracy of prediction strongly depends on linkage disequilibrium (LD) between QTL and marker loci, as well as co-segregation over multiple generations (Sun et al., 2016). The magnitude of linkage disequilibrium in aquaculture species is extensive (Jones et al., 2017b), but assumed LD in most simulation studies has been low in both population-wide and within-family studies (Toro et al., 2017). Therefore, accounting for realistic LD in optimization of breeding design in the aquaculture industry needs to be tailored for each species where known in order to accurately predict accuracy of genomic breeding values and their sustained utility in practice.

#### Genotype by Environment Interaction

Most simulated breeding studies have assumed common environmental rearing conditions and high similarity between selection environment and commercial environment, where progeny is produced (Calus and Veerkamp, 2011; Hely et al., 2013). However, in reality, this almost certainly will not be the case (particularly with disease), and the impact of G × E interactions could be substantial. Hence, simulation studies should attempt to model the impact of G × E on genomic selection and accuracies of selection and responses, as a function of genetic correlations between divergent environments, including long-term changes in environments such as predicted to occur through global warming for instance.

#### Bio-economical Modeling

Most of the simulation studies to date have focused on genetic gain and inbreeding however, the objective of the breeding program should be to maximize long-term profit. In that sense, cost and benefit analysis of genomic selection and profitability of the breeding program, particularly the additional cost of genotyping, should be evaluated using bio-economical modeling. This will include translating genetic gain and inbreeding on economic returns. Bio-economic modeling becomes more important in simulations when we realize that it has substantial effects on genetic improvement and efficiency of the selective breeding programs (Llorente and Luna, 2016).

## Practical Considerations for Implementing Genomic Selection on Farm

The greatest immediate value from genomic selection is realized where genomic breeding values can be targeted against traits that drive economic returns to commercial farmers. Typically, such traits are based on yields of harvested product. Although this sounds straightforward enough, practical limitations become immediately apparent in situations where traits under commercial grow-out conditions vary substantially from performance recording environments in often pathogen-free central nucleus breeding facilities (as used in specific pathogen free shrimp breeding programs, for instance). For most aquaculture systems, the G × E interactions are largely unknown and limit the value of genomic selection training data if the genetic correlation between the central nucleus breeding values and on-farm breeding values is significantly less than unity (i.e., < 0.6). Fortunately, genomic selection platforms allow for field data to be linked to nucleus broodstock through DNA derived genomic relationships and on-farm phenotyping. Second, genomic selection programs become increasingly more complex when harvest yields are determined by diverse genomes, as is the case of pearl oyster, with a host recipient seeded with the saibo of a donor. The need to have accurate breeding values for both host and donor oyster may eventually result in the need of separate breeding lines for both relatives to their contribution to pearl quality. Pearl formation in *P. maxima* and *P. margaritifera* appears to be influenced by environmental effects and genetic components of both the donor and host oyster (McGinty et al., 2012; Blay et al., 2017, 2018). The unclear genetic interactions between host and donor further complicate the application of genomic selection if such effects are significantly greater than zero. In the case of pearl oyster, the multi-factorial nature of pearl value adds to the complexity of setting up multi-trait genomic selection. Thirdly, and potentially of greatest commercial appeal for genomic selection is to build disease resistance into the genetic improvement program as has been highlighted above. Most central nucleus breeding programs are pathogen free, and breeding decisions are based on family sib selection, but commercial grow-out environments are under constant disease challenge. It is unlikely that simply screening commercial stocks will yield data of sufficient quality to obtain genomic breeding values for disease resistance, since most disease field challenges are uncontrolled, and often resistance to multiple pathogens is of interest. One potential solution is to expose large mixed-family progeny cohorts to standardized disease challenge and ascertain survival statistics from pooled genotype data pre- and post-challenge. Finally, it is almost certain that for most genomic selection programs, there will be a need for ongoing phenotyping to update the training sets and cross validate data collected under diverse commercial environments and to monitor unfavorable genetic correlated responses.

Perhaps one of the greatest advantages offered by the application of genomic selection over conventional breeding programs is that large-scale multi-family data can be resolved retrospectively through genomic relationships. This has two immediate and highly significant advantages. First, the predicted genetic response and realized inbreeding are far superior over the management of multiple single-family lines. Simple simulation shows that a cohort of 100 families in a single line outperforms the average of 100 single-family lines and creates the long-term sustainable value for the industry (Khatkar et al., 2017). Second, the enormous costs in establishing and maintaining single-family mating, spawning, and rearing facilities are not required under a genomic selection program using a large-scale multi-family breeding program. In many cases, the commercial infrastructure for propagation is sufficient, and the cost saving outweighs the cost incurred for genotyping.

In our experience, the transition from existing/traditional selection programs into a genomic selection program is challenging since most mating and infrastructure designs in central nucleus breeding facilities do not capture the advantages offered by genomic selection programs. In the case where simple mass produced, commercial stocks are produced, or where no genetic improvement programs are in place, imposing a genomic selection program is potentially straightforward. The main requirement is that the species is domesticated, since life cycles need to be closed for ongoing selection and capture of genetic gain. Where source broodstock has been harvested from wild stock, the base generation needs to be adequately represented in the foundation stocks, and inclusion of “new” ongoing sampling of wild stocks limited. Once an adequate training data set against commercially well-defined breeding objectives has been completed, a robust test-set and validation phase is required to determine the accuracy of the genomic predictions. For easy to measure traits of moderate-to-high heritability, this is relatively easy to achieve; however, for most, if not all diseases, and complex multi-factorial traits, the development of adequate training data sets will remain a logistical challenge. Of practical concern is also how best to use available information. For most applications, genotyping potential candidates under selection remains a significant cost. The use of multi-stage selection, based on simple phenotypic selection as a primary selection, followed by genomic sampling (DNA sampling genotyping and tracking tagged individuals) and selection is likely the most cost-effective application of this technology (Khatkar et al., 2017). Other applications of genomic selection include the genomic management to minimize inbreeding by candidate selection and mate allocation to maximize genomic diversity. Genomic selection also offers an additional commercial benefit, to pre-screen females and males in the current generation for the production of commercial animals, given that relatively few females are needed to generate the many millions of larvae for commercial production. The exact benefits of genomic selection breeding programs will be dependent on the species and nature of the aquaculture enterprise.

## Future Outlook for Shrimp and Pearl Oyster Genomic Selection Application

With rapid technological advances throughout the genomic selection pipeline, it is understandable that uptake by the industry is often lagging behind. This is also the case with emerging breeding programs in aquaculture in particular those that do not have a strong historical background in structured genetic improvement programs. In *L. vannamei*, the advances are significantly ahead of *P. monodon*, despite similarity in genomic resources, phenotyping, and broad principles of culture systems. A fundamental difference is in the stage of domestication, and being able to fully close life cycles between the species, which is a significant limitation for the adoption of advanced breeding systems in *P. monodon*. In pearl oyster the complexities of definition of breeding objectives, the relative importance of host and donor lines, which ultimately may require separate breeding programs, will challenge genomic selection programs in this production species. The importance of production focused outputs, versus key determinants that affect production efficiency, particularly disease outbreaks and production inputs (largely feed and labor requirements), will remain a technological challenge in defining appropriate multi-trait breeding objectives and selection programs for both shrimp and oyster. As phenotyping systems for disease resistance become better defined and manageable on a commercial scale, advanced selective breeding programs will become a better place to generate relevant genomic selection training populations and rapidly implement genomic breeding values to aid selection.

In the medium term, the continued collection of commercial phenotypes across a diverse range of environments combined with large-scale low-cost genotyping will provide avenues for systematic analysis and exploitation of G × E effects including the establishment of specialized breeding lines as well as the potential capture of non-additive effects through heterosis. The impact of genomic selection on the prediction of phenotypes within commercial production systems, and recording of detailed environmental effects right down to individual pond and hatchery effects, will see genomic information integrated in precision farming systems.

In the long term, incorporation of accurate and low-cost industrial-scale genotyping and on-farm phenotyping technologies will become the pipeline to generate data-rich resources matching genotype, by the way of full genome sequence, to extensive trait panels on many hundreds of thousands of animals phenotyped. This may allow QTL to be mapped down to quantitative trait nucleotide (QTN) polymorphisms as the causative determinants for such complex traits. Accordingly, such QTN could then be incorporated in genomic breeding value estimates, but ultimately it will open the way for alternative technologies such as CRISPR-Cas9 genome editing tools to enhance genetic improvement programs. In conclusion, the opportunities for genomic information to generate profitable and sustainable aquaculture systems for shrimp and oyster are currently at their infancy but with a real and potentially unprecedented return on investment for the near and long-term future.

## Author Contributions

All authors contributed significantly to the manuscript. The manuscript was conceived and prepared by KZ. Sections discussing practical considerations and future outlook were drafted by HR and DRJ. Authors MK, HR, and NK drafted the aspects relating to genomic selection accuracy, while DBJ added the quantitative trait content. DRJ and HR addressed the structural components within the manuscript. Overall manuscript clarity was reviewed by all authors, and all approved its content.

### Conflict of Interest Statement

The authors declare that the research was conducted in the absence of any commercial or financial relationships that could be construed as a potential conflict of interest.
